# Is vitamin C enough? A case report of scurvy in a five-year-old girl and review of the literature

**DOI:** 10.1186/s12887-019-1437-3

**Published:** 2019-03-08

**Authors:** Timothy Hahn, Whitney Adams, Keith Williams

**Affiliations:** Feeding Program, Penn State Hershey Medical Center, 905 W. Governor Road, Hershey, PA 17033 USA

**Keywords:** Vitamin C deficiency, Food selectivity, Nutrient deficiency, Anemia

## Abstract

**Background:**

Numerous cases of scurvy secondary to diet limitations have been reported in the literature with most being boys with special needs. To date, the focus of the literature describing vitamin C deficiency has been the medical sequelae of the deficiency. There has been little attention given underlying diet limitations causing the vitamin C deficiency.

**Case presentation:**

A five-year-old female with typical development initially presented with rash, then later for pain in both lower extremities. After evaluation revealed vitamin C deficiency, she was admitted into an intensive day treatment feeding program. A feeding assessment found she had life-long problems with eating and had a diet that never exceeded ten foods. Across the course of treatment, she learned to eat 29 new foods. At six-month follow-up her body mass index had increased from the 1st to the 61st percentile. At one-year follow-up her body mass index was at the 85th percentile. All sequalae of her deficiency resolved.

**Conclusions:**

This case is unusual as most reported studies describe males with special needs. The severity of her eating issues suggest providers may consider referral to allied health professionals to address diet limitations for both children identified with nutrient deficiencies as well as children whose selective eating places them at risk for nutritional deficiencies or problems with growth. The child we described was anemic, like 42% of children described in the case literature on scurvy and like 32% of the children in this literature, our patient was underweight. In the literature, comorbid nutrient deficiencies were reported in 22% of the scurvy case studies. We suggest vitamin C supplementation is a necessary component for addressing vitamin C deficiency, but insufficient for addressing the diet limitations causing the nutrient deficiency.

**Electronic supplementary material:**

The online version of this article (10.1186/s12887-019-1437-3) contains supplementary material, which is available to authorized users.

## Background

While scurvy has been described as very uncommon [1] or rare [2] in the pediatric population, a recent study examining the scurvy in a large pediatric health care facility identified 32 children with vitamin C deficiency over a five-year span [3]. Of these 32 cases, four children developed scurvy as the result of a lack of diet diversity, with three having the comorbid diagnosis of autism and one with intellectual disability [3]. In this sample, there were no children with typical development who developed scurvy from a primary dietary deficiency. Reviews of the literature have also found few children with typical development develop scurvy as the result of a limited diet. One review examining 18 cases of scurvy and reported only case of a boy with typical development whose limited diet resulted in scurvy [2] while second review examined 23 cases of scurvy associated with selective eating and found four boys and one girl with typical development [4]. A case series conducted at a metropolitan hospital found seven children with scurvy secondary to dietary insufficiency over a period of 18 years, all of whom had special needs, mostly autism spectrum disorder [5]. In the following case study, we describe scurvy in a five-year-old girl without special needs. After description of the case, we discuss the treatment of vitamin C deficiency in the context of both the current case and the existing literature on vitamin C deficiency. The child in this case study is unusual due to her gender and absence of special needs. This study is the first to examine the dietary aspects of vitamin C deficiency.

## Case presentation

A five-year-old typically developing female was referred by her primary care provider for evaluation of a rash reported to be sensitive to sunlight and had been reoccurring for the last 4 years. Cutaneous exam revealed fine scale on her trunk and extremities as well as small pink flat papules on lower left leg. She was diagnosed with ichthyosis vulgaris and provided with a treatment plan. She was subsequently referred to gastroenterology for decreased appetite. Two weeks later the child presented to the Emergency Department for right knee pain. Her mother reported her daughter had experienced bilateral lower extremity pain and had been limping. During the evaluation she was able to walk for the physician without reporting pain and no tenderness was reported with palpation. The mother reported her daughter was bitten by a tick about 1 year ago and that no workup or treatment was conducted. Radiographic scans of the right knee and leg showed no abnormalities. Laboratory testing for Lyme disease was conducted and found to be negative. She was discharged with the diagnosis of tenosynovitis. The child presented again to the Emergency Department the following week for left knee and ankle pain. She reported pain in her left calcaneus when asked to bear weight. Migratory arthralgia was noted and further laboratory testing, including a vitamin C level, was ordered. Her vitamin C level was found to be < 5 umol/L (reference range: 23 to 114 umol/L). Her prealbumin level was also low, 14 mg/dl (reference range: 17–36 mg/dl) as was both her ferritin 3.9 ng/ml (reference range: 6.2–137.0 ng/ml) and her iron saturation 15% (reference range: 20–55%). Both vitamin A and vitamin D were found to be within the normal reference range. With the diagnosis of the vitamin C deficiency, the child was started on ascorbic acid and referred to the feeding program.

The participant was the youngest of six children and raised by her biological parents in a middle-class household in a small town. She was born full term by caesarian section with a birth weight of 3.88 kg. There were no prenatal or postnatal complications. No delays in development were noted by the primary care provider or parents. She scooted at 6 months and walked before 1 year. No problems were noted with her gait until several weeks prior to the initial visit to the Emergency Department for knee pain.

**Fig. 1:**
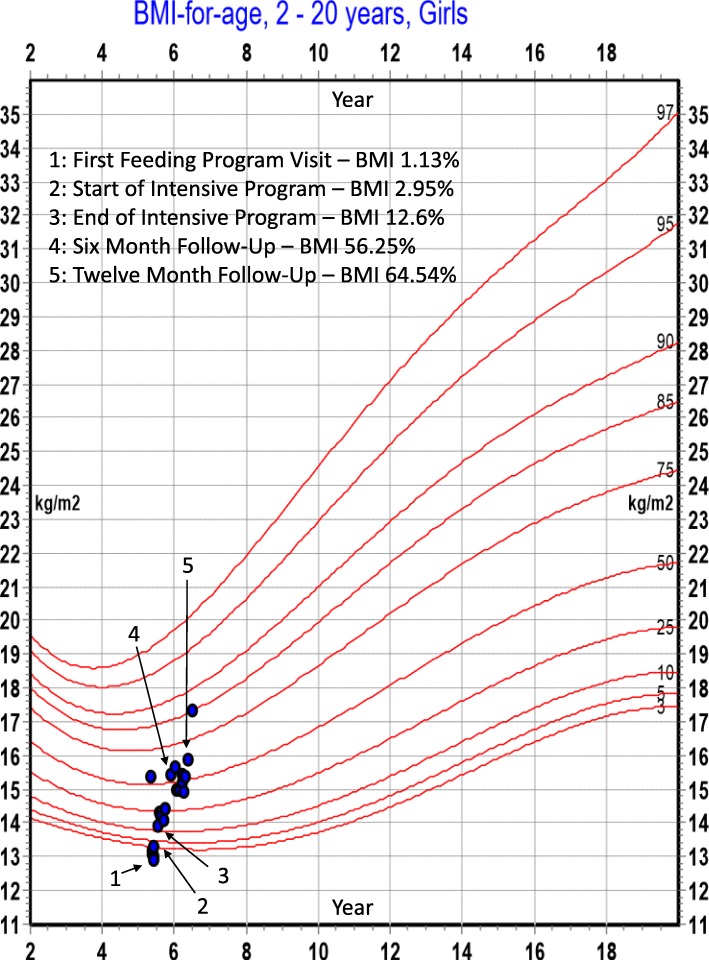
BMI percentile changes across treatment

At 1 year of age, she transitioned from infant formula to milk and cereal snacks. She never accepted baby food. For 4 years after transitioning off infant formula her diet consisted of cereal snacks, one type of cracker, and, inconsistently, chocolate pudding, vanilla ice cream, chocolate, and banana. Except for the occasional banana, she never ate fruit, vegetables, or meats. She drank skim milk, water, and, rarely, soda. She refused to taste new foods or drinks. Additionally, at the time of her diagnosis with vitamin C deficiency, her body mass index was at the 1st percentile. Based upon her inadequate growth and extremely limited diet, she was admitted to an intensive day treatment feeding program. During the course of intensive treatment she learned to eat 29 foods from all food groups through the use of an intervention involving gradual repeated exposure to novel foods [6]. In 6 months after intensive treatment, her weight had increased by four kilograms, her height increased by three centimeters, and her body mass index reached the 61st percentile. At 1 year after completion of intensive treatment, her height had increased by 7.6 cm and her weight had increased by seven kilograms Her body mass index reached the 85th percentile (see Fig. 1). Across the span of the 1 year after intensive treatment she continued to be monitored as an outpatient by a feeding therapist who continued to address the child’s diet variety and helped the family maintain the gains made during intensive treatment. At all outpatient appointments, a meal was conducted allowing the therapist to verify the child’s consumption of a variety of foods.

How unusual was this case?

To determine how this case compared to the existing literature, articles describing cases of scurvy secondary to diet limitations published from 2000 to 2018 were reviewed. PubMed and Google Scholar were searched using the search terms, “scurvy” and “ascorbic acid deficiency”. The reference section and citation listing of each article identified from these searches were then examined to identify additional articles. Sixty-one articles describing either a case study or a case series reported on 77 children diagnosed with scurvy as a result of diet insufficiencies (see Additional file 1 for an alphabetical listing of all studies). Selected demographics from these studies are shown in Table 1. While the child in the current study was only slightly younger than median age as the children in the existing literature, she differed from most of these children who were predominantly males with special needs, most often, autism spectrum disorder. Of the 77 reported cases, only six were females without special needs.

**Table 1:** Demographics of children diagnosed with scurvy

Age in years (*N* = 77)		
Mean	Median	Range
6.4	5	> 1–18
Total Sample (N = 77)		
Typical Development	Autism Spectrum	Other Special Needs______
28 (19 male)	25 (25 male)	24 (17 male)
Selective Eaters Only (*n* = 59)		
Typical Development	Autism Spectrum	Other Special Needs______
17 (14 male)	24 (24 male)	18 (13 male)

While all 77 children reported in these studies had limited diets, it is not clear that all could be described as selective eaters or to what degree their diets were the result of refusal to eat a variety of foods. Six of the children exhibited oral motor dysfunction which limited the range of foods they could eat [7, 8]. While some, or possibly all, of these six children might have obtained sufficient nutrition by consuming lower texture foods, oral supplement, or multi-vitamins, it is probable their parents did not know how to modify their diets to match their oral motor limitations. There were also three children dependent upon tube feeds who either received an insufficient amount of enteral formula [9] or received homemade tube feeds deficient in vitamin C [10]. There were other children whose diets were not adjusted to meet their specific health needs, for example, one child receiving a ketogenic diet was not receiving any vitamin supplementation [11] and another child received only a limited diet due to multiple food allergies [12]. For various reasons, the parents of 18 children, (five children with typical development, 14 children with special needs other than autism) limited their diets to the point these children developed vitamin C deficiency. Of the nine children with typical development with parent-limited diets, all but two were less than 2 years of age.

Fifty-nine of the children described in the existing literature could be described as selective eaters whose scurvy resulted from their limited intake. Of these children, 41% had autism spectrum disorder, 31% had intellectual disabilities, and 86% were male. Four of these 59 children were females with typical development like the girl in the current study. The child in the current case study had a diet similar to the diets of these 59 children identified as selective eaters in the existing literature on vitamin C deficiency. None of these children reportedly consumed vegetables or fruits, most consumed only starches and dairy products, with a few eating a limited number of proteins. The child in our case study was anemic, as were 42% of the children in the existing literature, an expected finding given the role of vitamin C in iron absorption. While the child in our case study did not exhibit signs or symptoms indicative of additional nutrient deficiencies, other deficiencies were considered likely so she was placed on a multivitamin within a day of the initial diagnosis of vitamin C deficiency. Her pediatrician conducted further testing and found her vitamin A and vitamin D levels were within the normal range. Of the 59 children described in the existing literature, 22% were identified with an additional nutrient deficiency beyond anemia (e.g. vitamin A, vitamin D). Given the diets reported for these 59 children, it is possible, if not probable, that more of these children had other nutrient deficiencies, but further deficiencies were either not reported or not identified.

The girl described in our case study had a body mass index at the 1st percentile prior to her feeding treatment. Underweight was also a common problem among the 59 children in the existing literature, with 32% being described as underweight. It is not surprising so many of these children were underweight. Many of children had anemia, which decreases appetite and eating was no doubt uncomfortable or even painful for many of these children, 71% of whom exhibited gingival symptoms.

## Discussion and conclusions

Every study in the existing literature on scurvy resulting from dietary insufficiency described the use of ascorbic acid to address the Vitamin C deficiency. In many instances, the case studies describe the rapid and almost complete resolution of all symptoms secondary to the vitamin deficiency. While we would not argue vitamin C supplementation does not resolve vitamin C deficiency, we do suggest vitamin C supplementation alone is insufficient for many children. For the 22% of the children with additional nutrient deficiencies, vitamin C alone would be inadequate for meeting their nutritional needs. While vitamin supplementation can, and does, correct nutrient deficiencies, supplementation does not correct the selective eating patterns of these children, which is the underlying reason the child in the current case study, and 59 children in the existing literature, developed scurvy. As demonstrated by the child in this case study, it is possible to address the underlying eating problems which led to vitamin deficiency. It is not known if the failure to address the eating problems underlying the nutritional deficiencies described in these 59 children puts them at risk for future nutritional deficiencies or problems with growth.

Research on children who exhibit more extreme patterns of selective eating have been shown to exhibit these patterns over prolonged periods of time [13]. There is no evidence that children who self-limit their intake to the extent they develop nutritional deficiencies will change their patterns of eating without intervention. Even though the presentation of a child with a vitamin C deficiency secondary to either selective eating or parents limiting the child’s diet may not be a common occurrence, it may also not be considered rare, with 61 published studies in the last 18 years. In our own healthcare organization, the current case was the fifth case of vitamin C deficiency in less than 5 years. Three other children presented to pediatric hematology for hematological complications and were subsequently diagnosed with scurvy secondary to diet limitations [14] and one child receiving homemade tube feedings presented with scurvy [10]. While the current study described a child presenting with a vitamin C deficiency and discussed the literature describing cases of scurvy in the pediatric population, nutrient deficiencies are not limited to vitamin C, other clinical presentations involving other vitamin deficiencies, including vitamin A [15], vitamin B_1_ [16], and vitamin D [17] have all been reported in children with limited diets. Even though the presentation of children with nutrient deficiencies may not be commonplace for most providers, children at-risk for nutrient deficiencies will be seen far more often. In a sample of 422 children referred to our feeding program, 95 ate ten or fewer foods [18].

While the case studies and case series in the existing literature all report resolution of the vitamin deficiency, the long-term outcomes of these children are unclear. Follow-up information from 6 months or longer after initial treatment of the vitamin C deficiency was reported for only nine children from the existing literature. Based upon our experience with selective eaters more generally, these children remain at risk for additional nutrient deficiencies or problems with weight, either underweight or possibly overweight.

The child in the current case study had a limited diet since being weaned, despite receiving regular pediatric care. In the existing literature, reports of contact with pediatric providers prior to diagnosis of the vitamin C deficiency were noted in numerous studies. There are no indications that children diagnosed with vitamin deficiencies are not receiving regular healthcare. Based upon our experience with the current case and the children referred to our organization’s feeding program, we suggest the extent of some children’s diet limitations are not always clear to healthcare providers. One large population-based study found 46% of parents identified their children as picky eaters at some point during childhood and picky remitted in two-thirds of cases within 3 years [19]. Picky eating does persist in some children, with one study showing picky eating as a stable trait through age 11 [20]. As providers hear about picky eating often and it usually resolves, it may be difficult to differentiate the transient picky eating commonly seen from the selective eating that could result in nutrient deficiencies.

While the role of dietary limitations on the development of nutritional deficiencies, namely vitamin C, was the focus of this case study and literature review, it is worth mentioning the child in this case study demonstrated a significant increase in her body mass index, increasing from the 1st to 85th percentile in 1 year. Certainly, some of this growth can be attributed to the increased number of calorie-dense foods she learned to eat, we also hypothesize the increased total variety of foods, including fruits and vegetables, also helped with weight gain. It is known that eating a food or limited foods over time results in monotony, or a decreased desire to eat this food or foods [21]. Increasing diet diversity can decrease the effects of monotony and lead to increased weight gain, especially if the diet contains some foods high in energy density [22]. Thus, increasing a child’s diet variety can not only prevent nutritional deficiencies, it can support adequate intake.

While the child in our case study was a young girl with typical development, our review of the clinical cases of vitamin C deficiency revealed children with special needs, especially children with autism spectrum disorders, were over-represented. This is consistent with the broader literature on childhood feeding problems which shows feeding problems occur at a higher prevalence in children with special needs [23]. As children with special needs are more at risk for long-term problems with feeding or eating, healthcare providers may provide additional attention to these children to determine the need for referral to providers to address feeding or eating problems.

## Additional file

Additional file 1: List of the 62 studies describing 77 cases of children with scurvy English. (DOCX 21 kb)
