# Correction to: Is vitamin C enough? A case report of scurvy in a five-year-old girl and review of the literature

**DOI:** 10.1186/s12887-019-1525-4

**Published:** 2019-05-16

**Authors:** Timothy Hahn, Whitney Adams, Keith Williams

**Affiliations:** Feeding Program, Penn State Hershey Medical Center, 905 W. Governor Road, Hershey, PA 17033 USA

**Correction to: BMC Pediatr**

**https://doi.org/10.1186/s12887-019-1437-3**

Following publication of the original article [1], the authors reported that an out-of-date version of Figure 1 had been incorporated in the published article.

Please see here for the updated (correct) version of the figure:

**Fig. 1:**
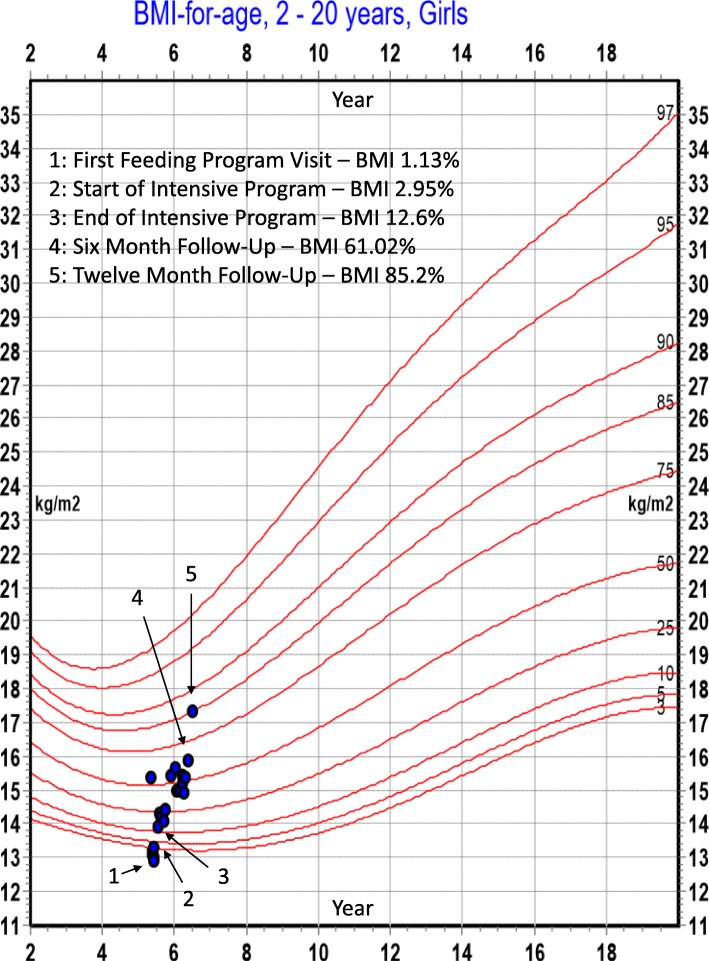
BMI percentile changes across treatment

The figure in the original article has been corrected accordingly.
