# Supplementary material for: Is vitamin C enough? A case report of scurvy in a five-year-old girl and review of the literature
Source: BMC Pediatr. 2019 Mar 8;19:74. doi: 10.1186/s12887-019-1437-3 (PMC6408840; doi:10.1186/s12887-019-1437-3)
Supplement: Supplementary file 1 — List of the 62 studies describing 77 cases of children with scurvy English. (DOCX 21 kb) [file 12887_2019_1437_MOESM1_ESM.docx]

Additional file 1. Cases of Scurvy 2000-2018

Akikusa JD, Garrick D, Nash MC. Scurvy: Forgotten but not gone. J Paediatr Child Health. 2003;39:75-77.

Alqanatish JT, Alqahtani F, Alsewairi WM, Al-Kenaizan S. Childhood scurvy: An unusual cause of refusal to walk in a child. Pediatr Rheumatol. 2015; 13:23.

Amos LE, Carpenter SL, Hoeltzel MF. Lost at sea in search of a diagnosis: A case of unexplained bleeding. Pediatr Blood Cancer. 2016; 63:1305-1306.

Aroojis AJ, Gajjar SM, Johari AN. Epiphyseal separations in spastic cerebral palsy. J Pediatr Orthop. 2007; 16:170-174.

Bacci C, Sivolella S, Pellegrini J, Favero L, Berengo M. A rare case of scurvy in an otherwise healthy child: Diagnosis through oral signs. Pediatr Dent. 2010; 32:536-538.

Bari A, Javaid B, Rehman S, Naz S. Scurvy: Presenting as musculoskeletal pain. J Coll Phys Surg Pakistan. 2009; 19:198-200.

Besbes LG, Haddad S, Ben Meriem C, Golli M, Najjar MF, Guediche MN. Infantile scurvy: Two case reports. Int J Pediatr. 2010; doi:10.1155/2010/717518.

Bingham AC, Kimura Y, Imundo L. A 16-year-old boy with purpura and leg pain. J Pediatr 2003;142:560-563.

Brambilla A, Pizza C, Lasagni D, Lachina L, Resti, M, Trapani S. Pediatric scurvy: When contemporary eating habits bring back the past. Front Pediatr.2018; 6.

Brennan CM, Atkins KA, Druzgal CH, Gaskin CM. Magnetic resonance imaging appearance of scurvy with gelatinous bone marrow transformation. Skel Radiol. 2012; 41:357-360.

Burk CJ, Molodow R. Infantile Scurvy. Am J Clin Derm. 2007; 8:103-106.

Bursali A, Gursu S, Gursu A, Yildirim T. (2009). A case of infantile scurvy treated only with vitamin C: A forgotten disease. Acta Orthop Belg. 2009; 75:428.

Cain M, Harris M. Kim K, Homme JH. Ascorbic acid deficiency (scurvy) in a toddler with restricted dietary intake presenting with “leg weakness” and a rash. Infant Child Adolesc Nutrit. 2014; 6:201-204.

Choi SW, Park SW, Kwon YS, Oh IS, Lim MK, Kim WH, Suh CH. MR imaging in a child with scurvy: A case report. Korea J Radiol. 2007; 8:443-447.

Cole JA, Warthan MM, Hirano SA, Gowen CW, Williams JV. Scurvy in a 10‐Year‐Old Boy. Pediatr Dermatol. 2011; 28:444-446.

Desai VD, Hegde S, Bailoor DN, Patil N. Scurvy extinct? Think again! Int J Clin Pediatr Dent. 2009; 2:39.

Duggan CP, Westra SJ, Rosenberg AE. (2007). A 9-year-old boy with bone pain, rash, and gingival hypertrophy. New Engl J Med. 2007;357:392-400.

Duvall MG, Pikman Y, Kantor DB, Ariagno K, Summers L, Sectish TC, Mullen, MP. Pulmonary hypertension associated with scurvy and vitamin deficiencies in an autistic child. Pediatr. 2013; 32:E1699-1703.

Erdle S, Conway M, Weinstein M. A six-year-old boy with autism and left hip pain. Can Med Asso J. 2017; 189:E275.

Estienne M, Bugiani M, Bizzi A, Granata T. Scurvy hidden behind neuropsychiatric symptoms. Neurol Sci. 2011; 32:1091-1093.

Gongidi P, Johnson C, Dinan D. Scurvy in an autistic child: MRI findings. Pediatr Radiol. 2013; 43:1396-1399.

Gulko E, Collins LK, Murphy RC, Thornhill BA, Taragin BH. (2015). MRI findings in pediatric patients with scurvy. Skel Radiol. 2015; 44:291-297.

Gupta S, Kanojia R, Jaiman A, Sabat D. Scurvy: An unusual presentation of cerebral palsy. World J Orthoped. 2012;3:58.

Harknett KM, Hussain SK, Rogers MK, Patel NC. Scurvy mimicking osteomyelitis: Case report and review of the literature. Clin Pediatr. 2014; 53:995-999.

Jacobsen A, DeNiro K. Rash and arthralgias in a teenager with autism.  JAMA Pediatr. 2017; 171:89-90.

Kinlin LM, Blanchard AC, Silver S, Morris SK. Scurvy as a mimicker of osteomyelitis in a child with autism spectrum disorder. Int J Infect Dis. 2018; 69:99-102.

Kitcharoensakkul M, Schulz CG, Kassel R, Khanna G, Liang S, Ngwube A, Baszis KW, Hunstad DA, White AJ. Scurvy revealed by difficulty walking: three cases in young children. J Clin Rheumatol. 2014; 20:224-228.

Küçükaydın Z, Dursun İ, Daldaban B, Özcan A, Ünal E. Scurvy: A rare cause of arthritis in a child with neurologic disorder. Eur J Rheumatol. 2018;10

Larralde M, Santos Muñoz A, Boggio P, DiGruccio V, Weis I, Schygiel A. Scurvy in a 10‐month‐old boy. Int J Dermatol. 2007; 46:194-198.

Ma NS, Thompson C. Weston S. Brief report: Scurvy as a manifestation of food selectivity in children with autism. J Autism Dev Disord. 2016;46:1464-1470.

Mawson AR. (2009). Bone pain, growth failure, and skin rash after an upper respiratory illness in a boy with autism: Possible association with altered retinoid metabolism. Clin Pediatr. 2009;48:21-25.

Narchi H, Thomas M. A painful limp. J Paediatr Child Health. 2000; 36:277-278.

Neri S, Pollicino C, Rizzotto A, Arcidiacono E. A forgotten disease in the land of citrus fruit. Nutritional Disorders Therapy 2012;2:106.

Niwa T, Aida N, Tanaka Y, Tanaka M, Shiomi M, Machida J. (2012). Scurvy in a child with autism: Magnetic resonance imaging and pathological findings. J Pediatr Hematol Oncol. 2012;34:484-487.

Noble JM, Mandel A, Patterson MC. Scurvy and rickets masked by chronic neurologic illness: Revisiting “psychologic malnutrition”. Pediatrics. 2007;119:e783-e790.

Noordin S, Baloch N, Salat MS, Memon AR, Ahmad T. Skeletal manifestations of scurvy: A case report from Dubai. Case Rep Orthop. 2012, doi:10.1155/2012/624628

Perry ME, Page N, Manthey DE, Zavitz JM. Scurvy: Dietary discretion in developed country. Clin Pract Case Emerg Med. 2018;2:147.

Polat AV, Bekci T, Say F, Bolukbas E, Selcuk MB. Osteoskeletal manifestations of scurvy: MRI and ultrasound findings. Skelet Radiol. 2015;44:1161-1164.

Popovich D, McAlhany A, Adewumi AO, Barnes MM. Scurvy: Forgotten but definitely not gone. J Pediatr Health Care. 2009; 23:405-415.

Riepe FG, Eichmann D, Oppermann HC, Schmitt HJ, Tunnessen WW. Picture of the month. Arch Pediatr Adolesc Med. 2001;155:607-608.

Rosati P, Boldrini R, Devito R, Menditto A, Barbuti D, Nibbi F, Mancini S, Reale A, Vignati E, Romanini M, Iannelli M, Marchili MR, Fierimonte V, Castelli M, Vitale L, Villani A. A child with painful legs. The Lancet. 2005;365:1438.

Rumsey DG, Rosenberg AM. Childhood scurvy: A pediatric rheumatology perspective. J Rheumatol. 2013;40:201-202.

Saha P, Pal RB, Das I, Sinha MK. Bilateral proptosis in a child with vitamin C deficiency. Int Ophthalmol. 2012;32:599-601.

Seya M, Handa A, Hasegawa D, Matsui T, Nozaki T. Scurvy: From a selective diet in children with developmental delay. J Pediatr. 2016; 177:331.

Sobotka SA, Deal SB, Casper TJ, Booth KVP, Listernick RH. (2014). Petechial rash in a child with autism and Trisomy 21. Pediatr Ann. 2014;43:224-226.

Solanki M, Baweja DK, Patil SS, Shivaprakash PK. Ascorbic acid deficiency: A case report. J Dent Child. 2011;78:115-119.

Srinivasan S. “I don't want to brush my teeth!” The case of an 8-year-old with gingival bleeding. Clin Pediatr Emerg Med. 2013;14:64-70.

Tamura Y, Welch DC, Zic JA, Cooper WO, Stein SM, Hummell DS. (2000).

Scurvy presenting as painful gait with bruising in a young boy. Arch Pediatr Adolesc Med. 2000; 154: 732-735.

Uda K, Goto M, Nigo A, Kono T. Early diagnosis of scurvy based on radiology and dietary history. Pediatr Int. 2017;59:357-358.

Valentini D, Barbuti D, Grandin A, De Horatio LT, Villani A. A good growth in a child with scurvy. BMJ Case Rep. 2011;bcr1020103383.

Verma S, Sivanandan S, Aneesh MK, Gupta V, Seth R, Kabra, S. Unilateral proptosis and extradural hematoma in a child with scurvy. Pediatr Radiol. 2007;37:937-939.

Vitale A, Torre FL, Martini G, Calcagno G, Fede C, Conti G, Chimenz R, Zulian F. Arthritis and gum bleeding in two children. J Paediatr Child Health. 2009;45:158-160.

Vitoria I, López B, Gómez J, Torres C, Guasp M, Calvo I, Dalmau J. (2016). Improper use of a plant-based vitamin C–deficient beverage causes scurvy in an infant. Pediatr. 2016;137:1-5.

Weinstein M, Babyn P, Zlotkin S. An orange a day keeps the doctor away: Scurvy in the year 2000. Pediatr. 2001;108:e55-e55.

Willmott NS, Bryan RAE. (2008). Scurvy in child with epilepsy on a ketogenic diet with oral complications. Eur Arch Paediatr Dent. 2008;9:148-152.

Yan A, Conway M, Beck CE. Limp in a child with autism spectrum disorder. Global Pediatr Health. 2017;4:1-3.

Yee YC, Ying TP, Wei NS. (2017). The oldest nutritional deficiency disease: A case report of scurvy. Malaysian J Paediatr Child Health. 2017;22:41-45.
